# Invited commentary: “Identifying traumatic significant haemorrhage is challenging for patient with low and intermediate risk, not when bleeding is obvious”

**DOI:** 10.1186/s13049-023-01162-1

**Published:** 2023-12-12

**Authors:** Joanne E Griggs, Richard M Lyon, Martyn Sherriff, Jack W Barrett, Gary Wareham, Ewoud Ter Avest

**Affiliations:** 1 Air Ambulance Charity Kent Surrey Sussex, Hanger 10 Redhill Aerodrome, Redhill, RH1 5YP UK; 2 School of Health Sciences, University of Surrey, Priestley Rd, Guildford, GU2 7YH UK; 3 Bristol Dental School, Faculty of Health Sciences, University of Bristol, Child Dental Health, Lower Maudlin Street, Bristol, BS1 2LY UK; 4 South East Coast Ambulance NHS Foundation Trust, Neptune House, Gatwick, RH10 9BG Surrey UK; 5 Department of Emergency Medicine, University Medical Center Groningen, Groningen, The Netherlands

We would like to thank the authors for their valuable comments on our study, wherein we investigated how pre-hospital lactate (P-LACT) measurements could be used to predict the need for (ongoing) in-hospital blood product transfusion in patients attended by HEMS with major traumatic haemorrhage.

As mentioned in our article, the algorithm we developed is a decision *support* tool, which means that it should be used in conjunction with other parameters, such as clinical gestalt in a heuristic approach to estimate transfusion requirements. The cut-off value of a P-LACT < 2.5 mmol/l used in our population yielded a sensitivity of 80% (corresponding to a low probability of major haemorrhage as the authors rightly mention), and hence was inadequate to be used in isolation. The SOP in our service states that a P-LACT < 2.5 mmol/l is used in conjunction with an SBP > 100mmHg to identify patients who have a low probability of major hemorrhage. This is supported by a recent publication of Gaessler et al. (2023) wherein the authors show that P-LACT and SBP are complimentary in terms of predictive probability [1].

To identify patients with a high likelihood of major haemorrhage requiring in-hospital transfusion, a P-LACT of 6.0 mmol/l was used, as at this this point the predicted probability curve (Fig. 2 in our original article) starts to flatten: using a higher cut-off would not have yielded a higher specificity, whereas a lower cut-off would have dropped specificity whilst not yielding a much higher proportion of the population meeting the cut-off criteria (n = 13, 6.7% for a lactate of 6.0 mmol/l vs. n = 17, 8.7% for a lactate of 5.5 mmol/l). Although we agree that it is likely that many patients with a lactate > 6.0 mmol/l will show clinical signs of shock, 5/13 patients had an SBP > 100 mmHg on first occasion, two of whom also did not exhibit tachycardia. In these patients P-LACT may still be a useful tool. Despite this however, the major challenge remains to identify the bleeding patients in the P-LACT group of 2.5-6 mmol/l, and serial measurements may be the way forward in this group.

Finally, we acknowledge that transfusion requirement is not always a good surrogate to use for outcome, especially not when confounding by indication may be present: using lactate may result in transfusing more patients in the pre-hospital setting, which again may result in a lower threshold to continue transfusion in-hospital. However, as 2/3 of the patients in our cohort received a massive transfusion (> 10 units PRBC within 24 h) rather than a major transfusion, we think transfusion requirement was a reasonable surrogate for risk of death from bleeding in our population. We agree however, that ideally outcome studies should be performed using hard endpoints to confirm this.

## Data Availability

Not applicable.
